# Supplementary figures and images for: Spatial and Texture Analysis of Root System distribution with Earth mover’s Distance (STARSEED)
Source: Plant Methods. 2023 Jan 5;19:2. doi: 10.1186/s13007-022-00974-z (PMC9814335; doi:10.1186/s13007-022-00974-z)

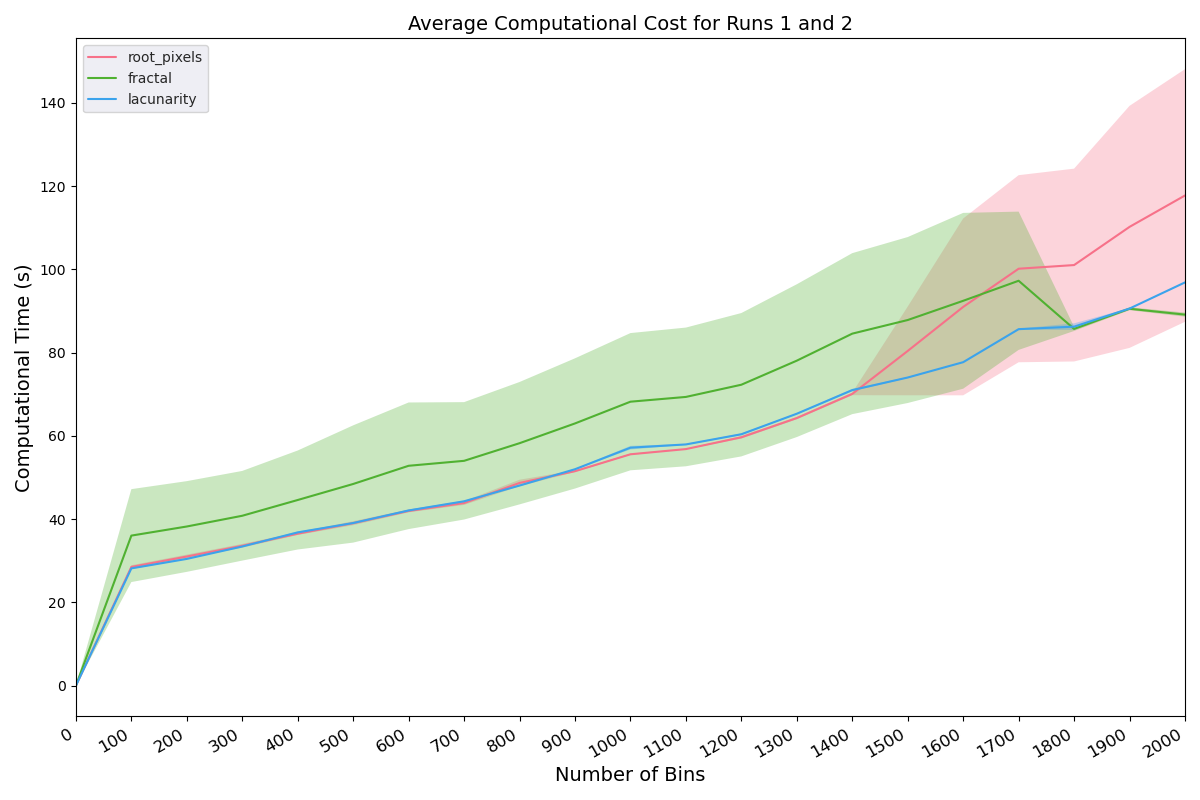

Supplement: Supplementary file 1 — Additional file 1: Fig. S1: Average computational cost for Runs 1 and 2. The shaded areas correspond to ± 1 standard deviation across three experimental runs of each feature and grid size value. The experiments were performed on a Dell XPS 15 9520 laptop with 12th Generation Intel Core i9-12900 HK processor. [file 13007_2022_974_MOESM1_ESM.png]

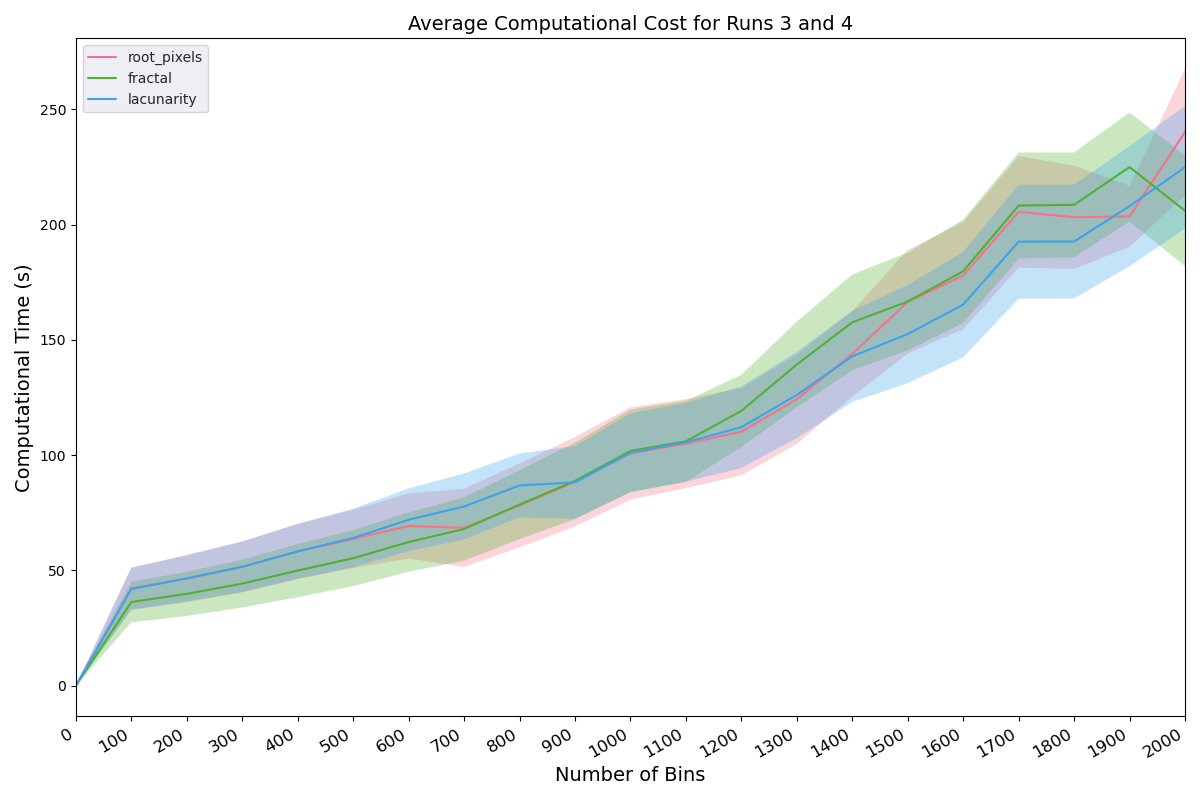

Supplement: Supplementary file 2 — Additional file 2: Fig. S2: Average computational cost for Runs 3 and 4. The shaded areas correspond to ± 1 standard deviation across three experimental runs of each feature and grid size value. The experiments were performed on a Dell XPS 15 9520 laptop with 12th Generation Intel Core i9-12900 HK processor. [file 13007_2022_974_MOESM2_ESM.png]

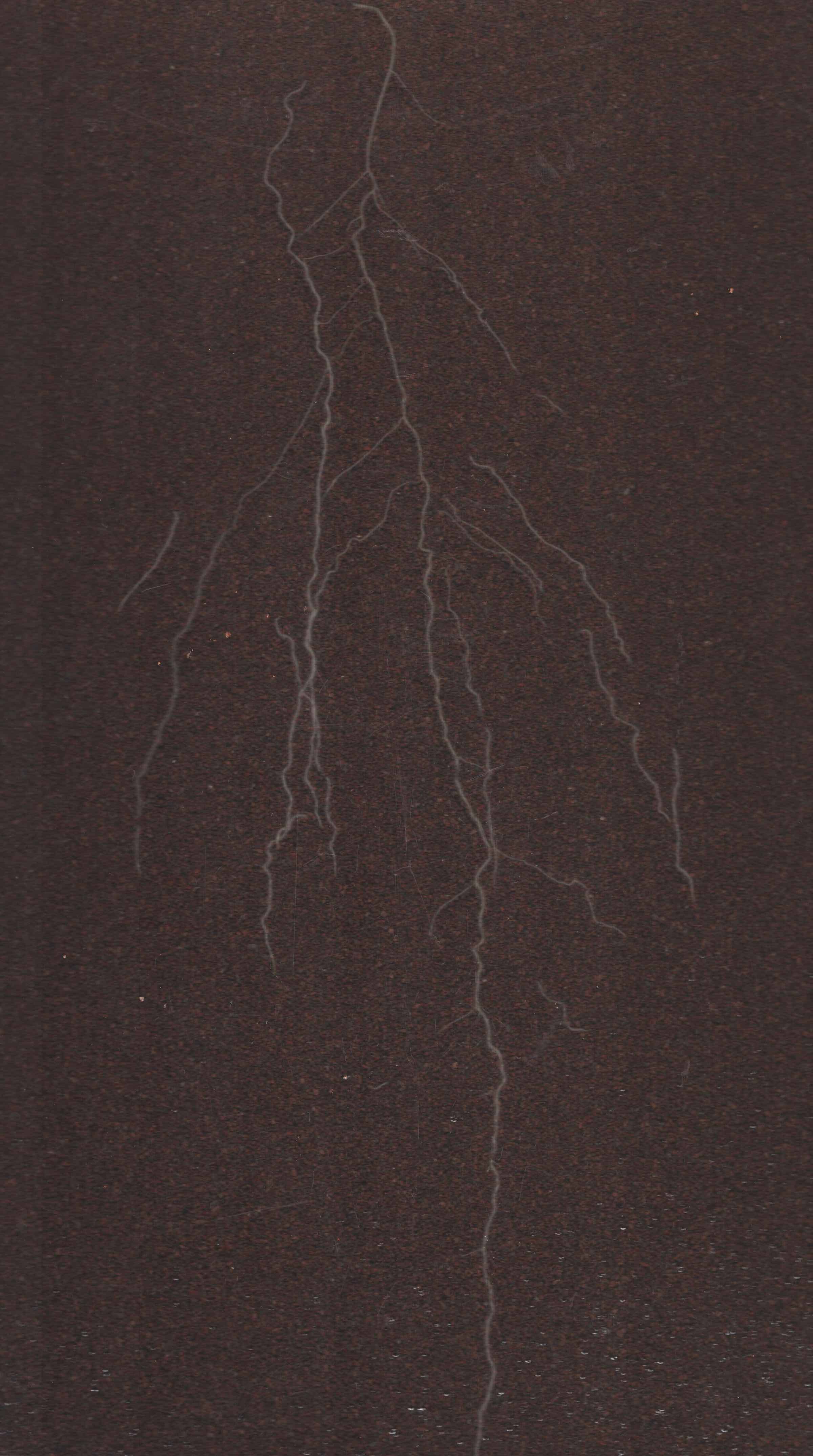

Supplement: Supplementary file 3 — Additional file 3: a) Cultivar S32 (b) Cultivar S35 (c) Cultivar S38 (d) Cultivar S40 Fig. S3: RGB root images of different cultivars with the same treatment of 60% water level. [file 13007_2022_974_MOESM3_ESM.zip › FigureS3a.jpg]

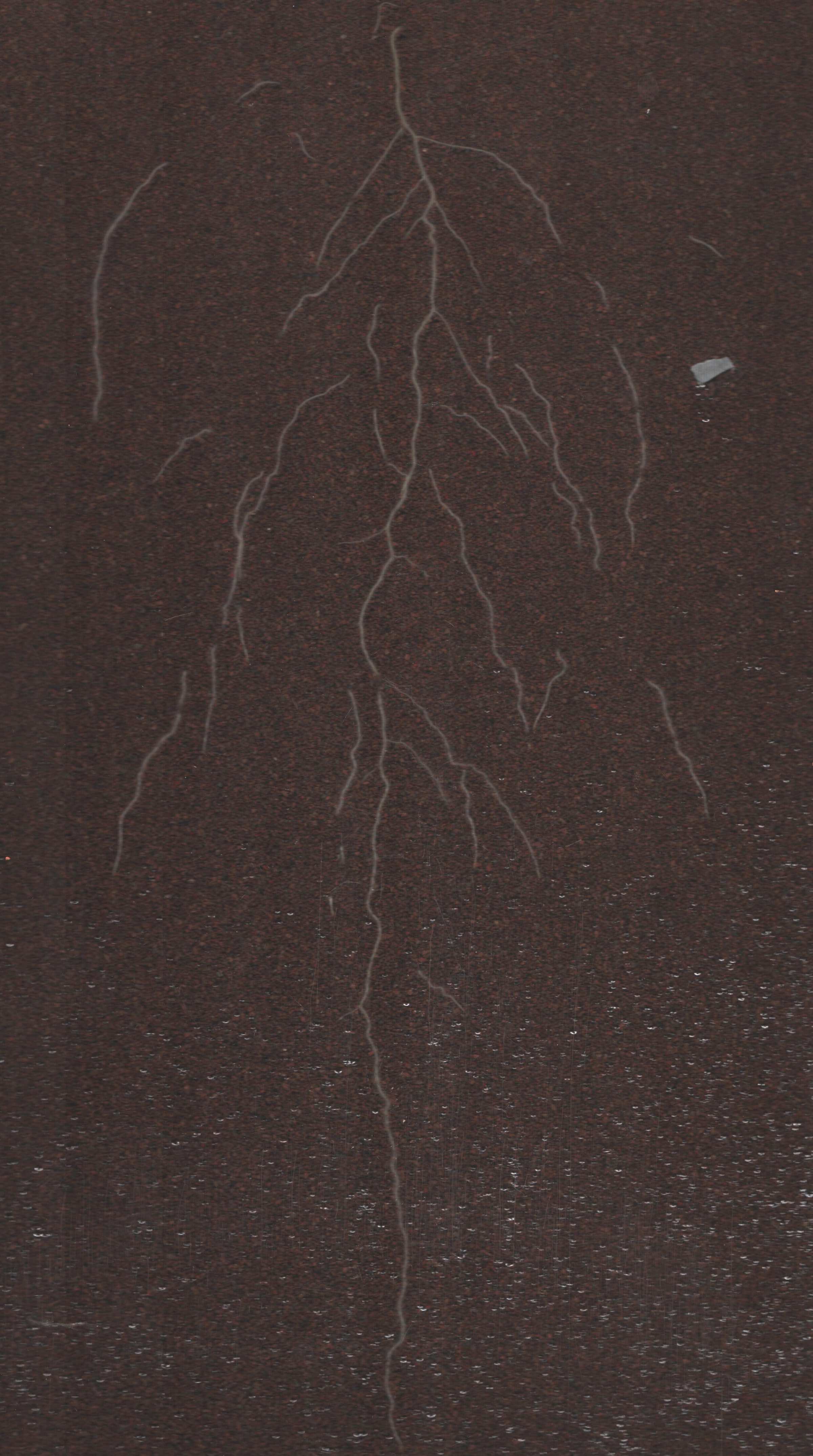

Supplement: Supplementary file 3 — Additional file 3: a) Cultivar S32 (b) Cultivar S35 (c) Cultivar S38 (d) Cultivar S40 Fig. S3: RGB root images of different cultivars with the same treatment of 60% water level. [file 13007_2022_974_MOESM3_ESM.zip › FigureS3b.jpg]

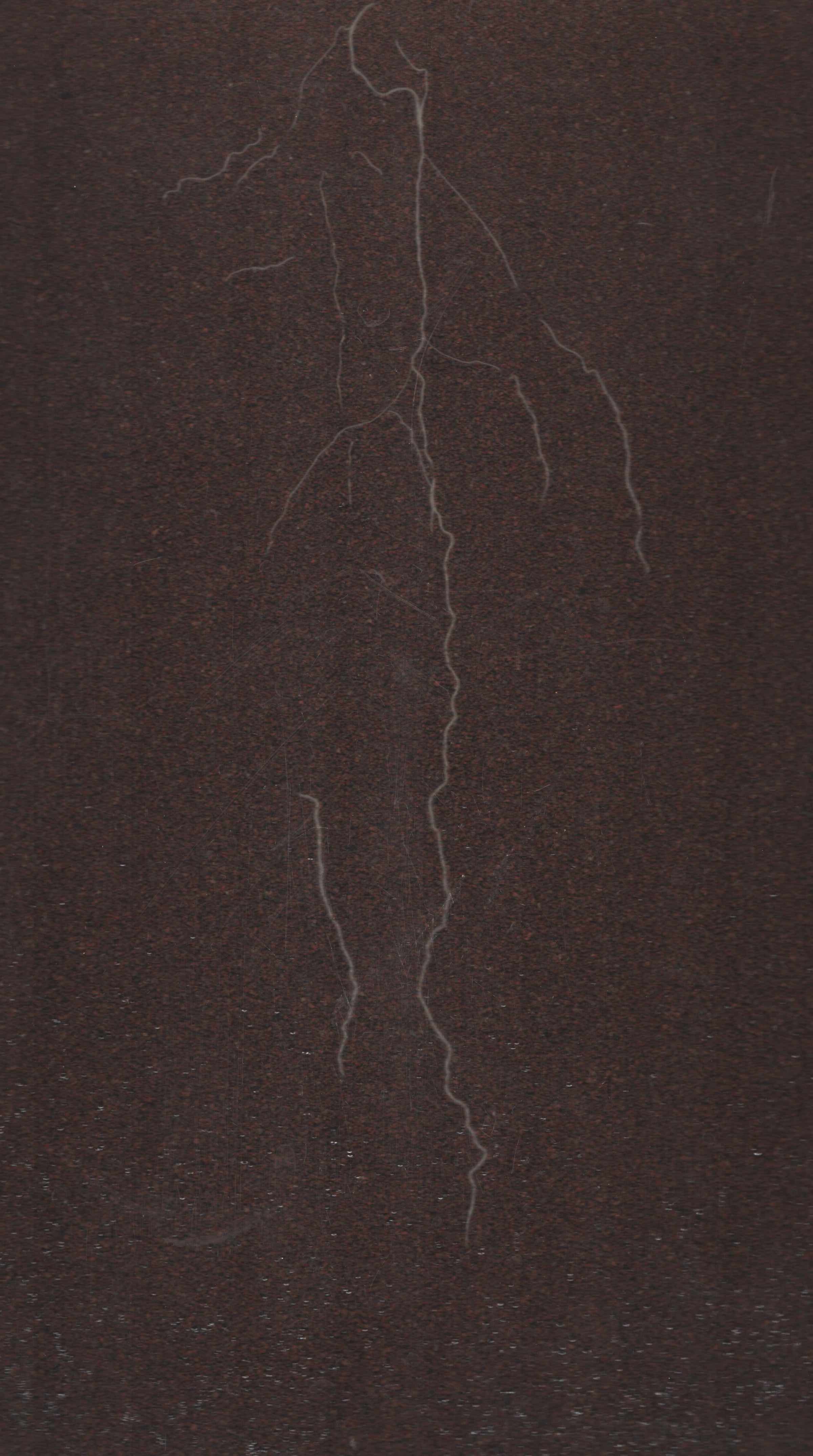

Supplement: Supplementary file 3 — Additional file 3: a) Cultivar S32 (b) Cultivar S35 (c) Cultivar S38 (d) Cultivar S40 Fig. S3: RGB root images of different cultivars with the same treatment of 60% water level. [file 13007_2022_974_MOESM3_ESM.zip › FigureS3c.jpg]

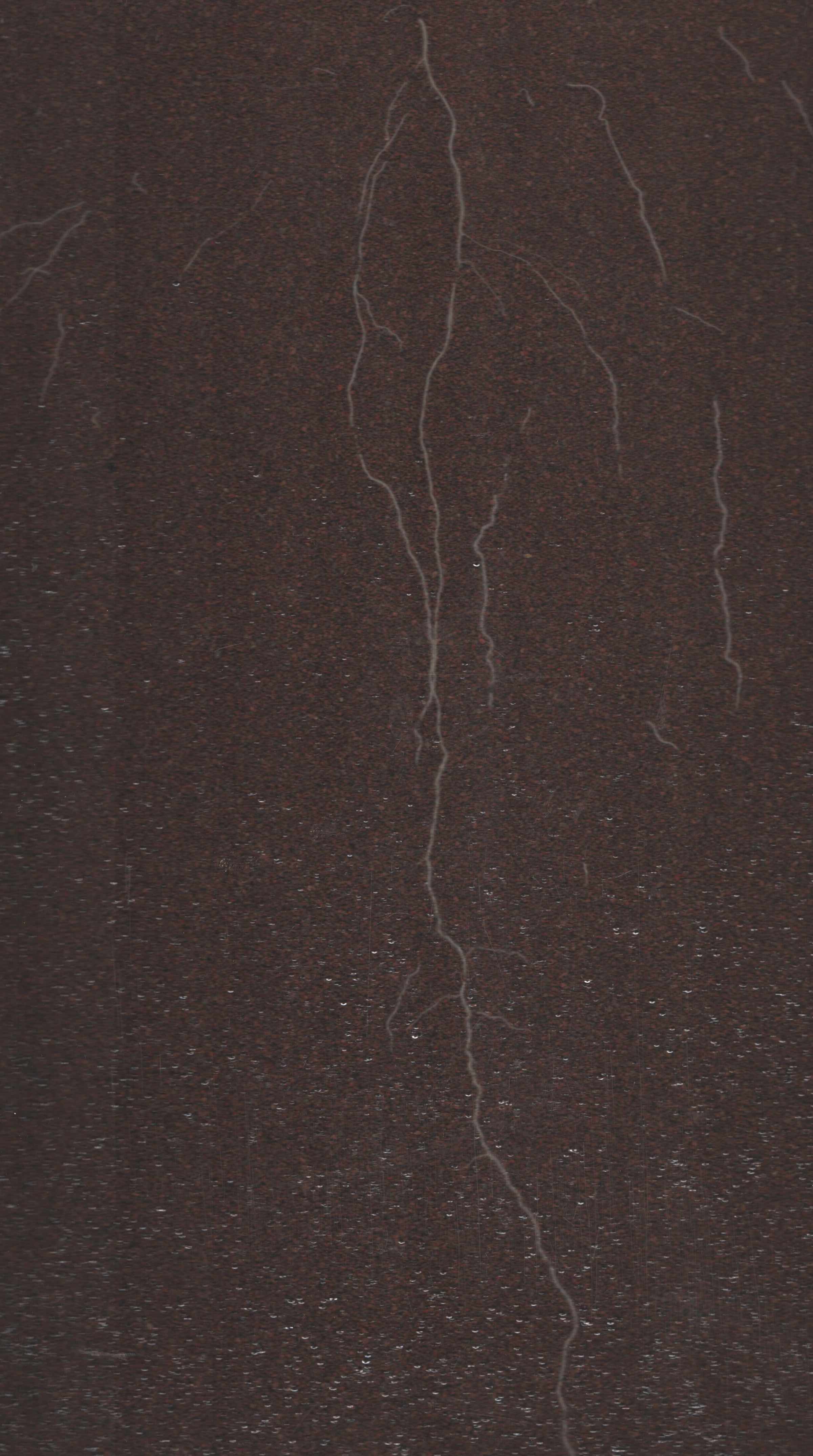

Supplement: Supplementary file 3 — Additional file 3: a) Cultivar S32 (b) Cultivar S35 (c) Cultivar S38 (d) Cultivar S40 Fig. S3: RGB root images of different cultivars with the same treatment of 60% water level. [file 13007_2022_974_MOESM3_ESM.zip › FigureS3d.jpg]

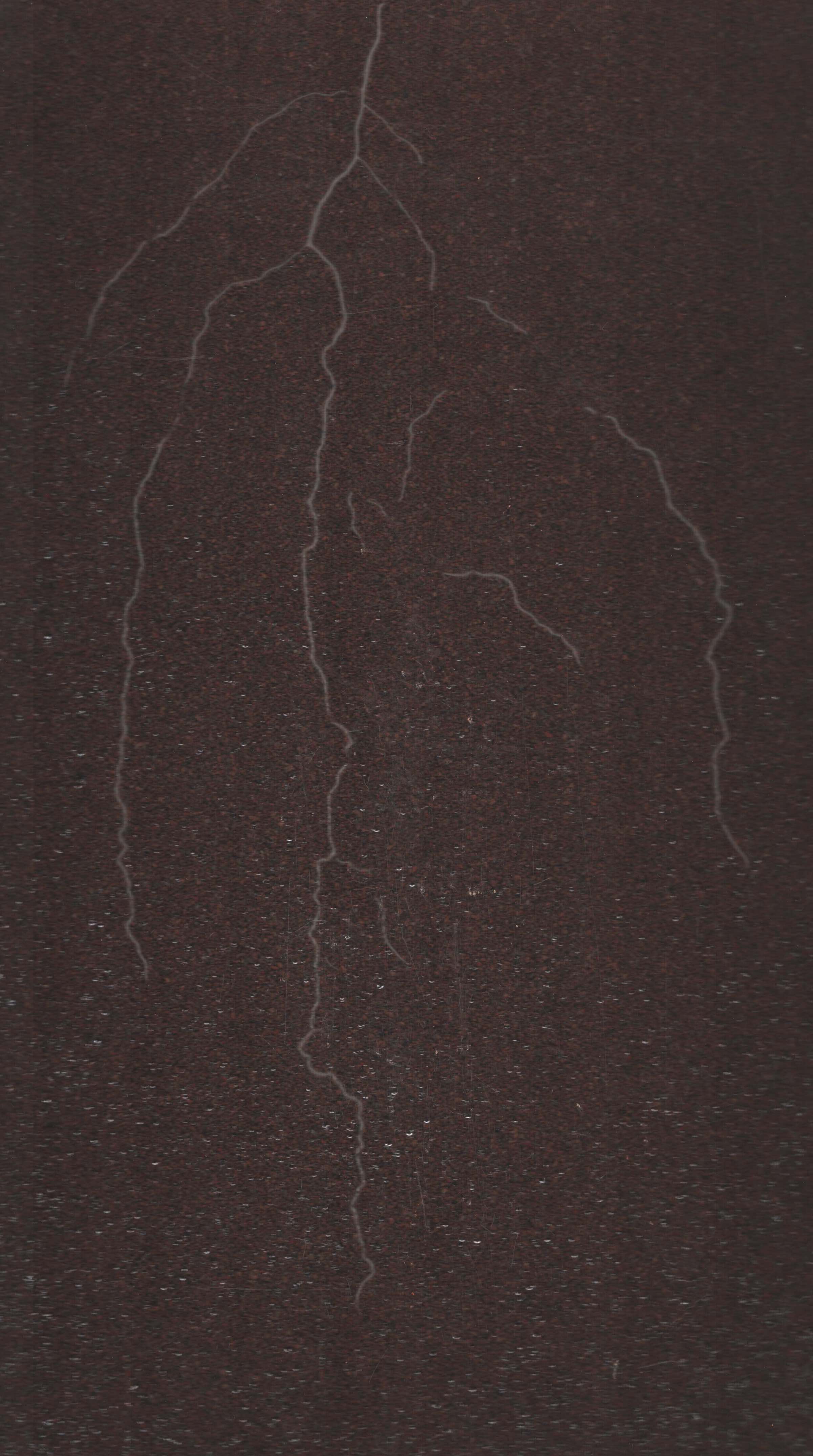

Supplement: Supplementary file 4 — Additional file 4: (a) Water level 60% (b) Water level 80% (c) Water level 100% (d) Water level 120% Fig. S4: RGB root images of the same cultivars (S38) with different water level treatments. [file 13007_2022_974_MOESM4_ESM.zip › FigureS4a.jpg]

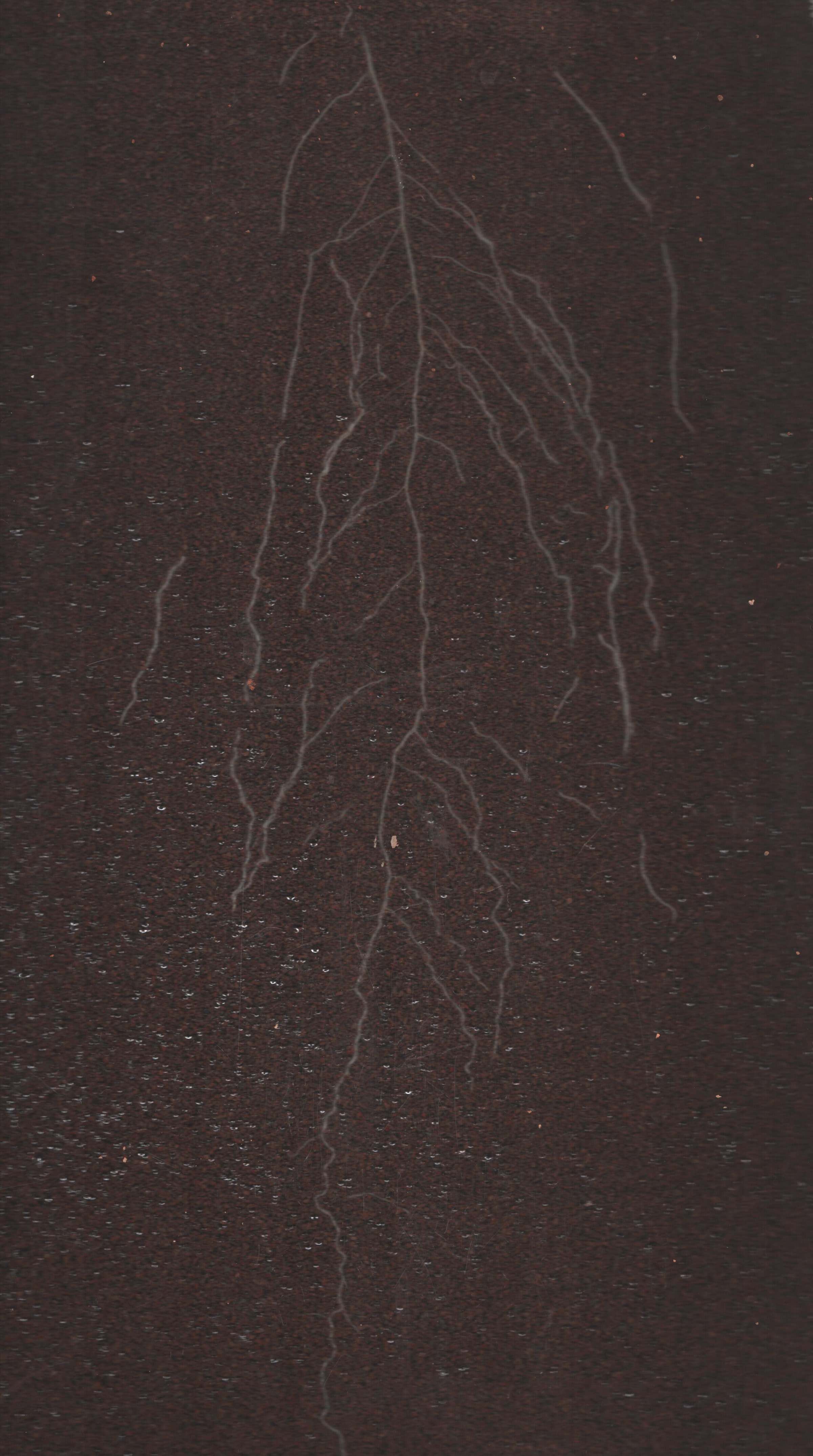

Supplement: Supplementary file 4 — Additional file 4: (a) Water level 60% (b) Water level 80% (c) Water level 100% (d) Water level 120% Fig. S4: RGB root images of the same cultivars (S38) with different water level treatments. [file 13007_2022_974_MOESM4_ESM.zip › FigureS4b.jpg]

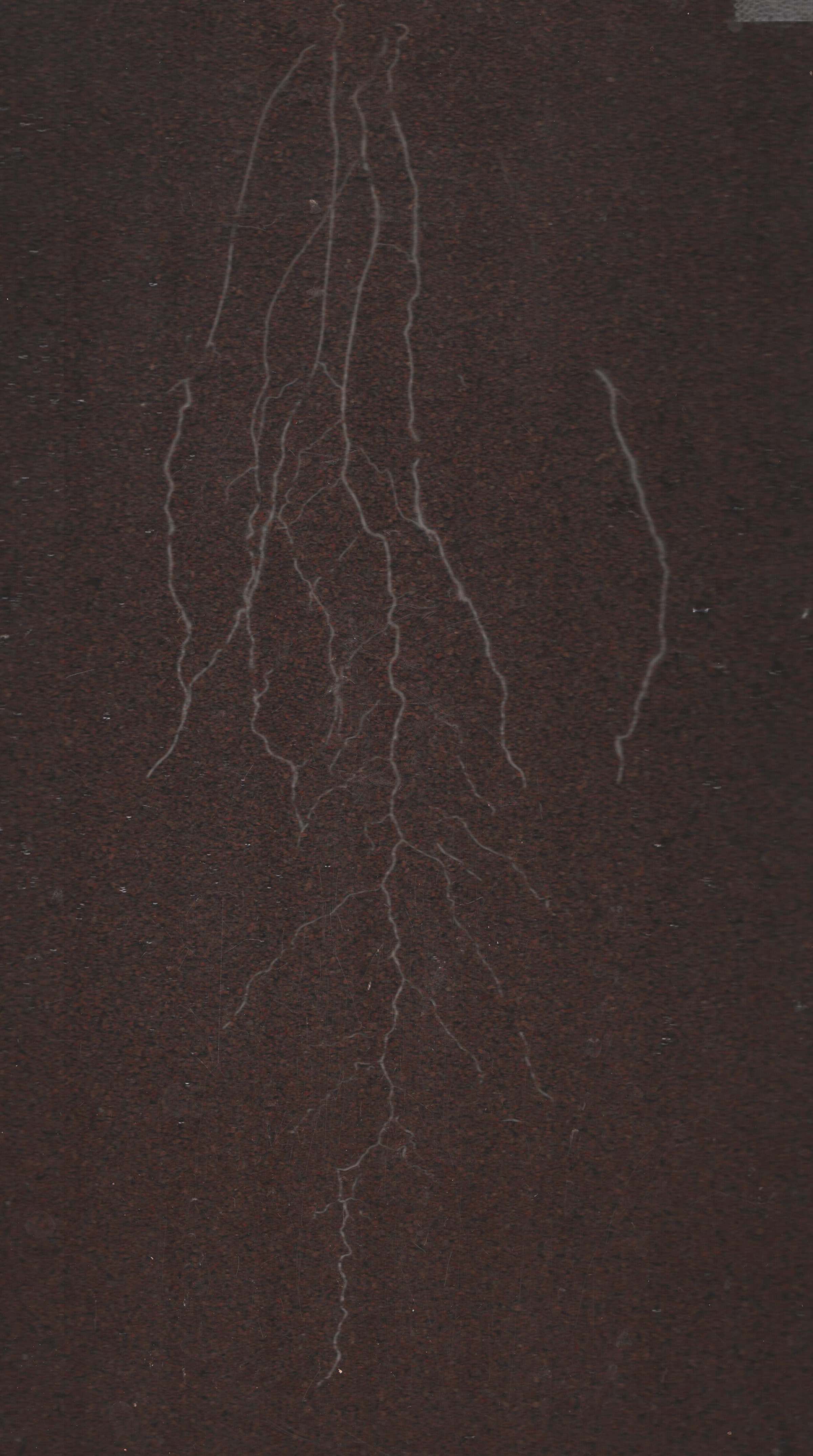

Supplement: Supplementary file 4 — Additional file 4: (a) Water level 60% (b) Water level 80% (c) Water level 100% (d) Water level 120% Fig. S4: RGB root images of the same cultivars (S38) with different water level treatments. [file 13007_2022_974_MOESM4_ESM.zip › FigureS4c.jpg]

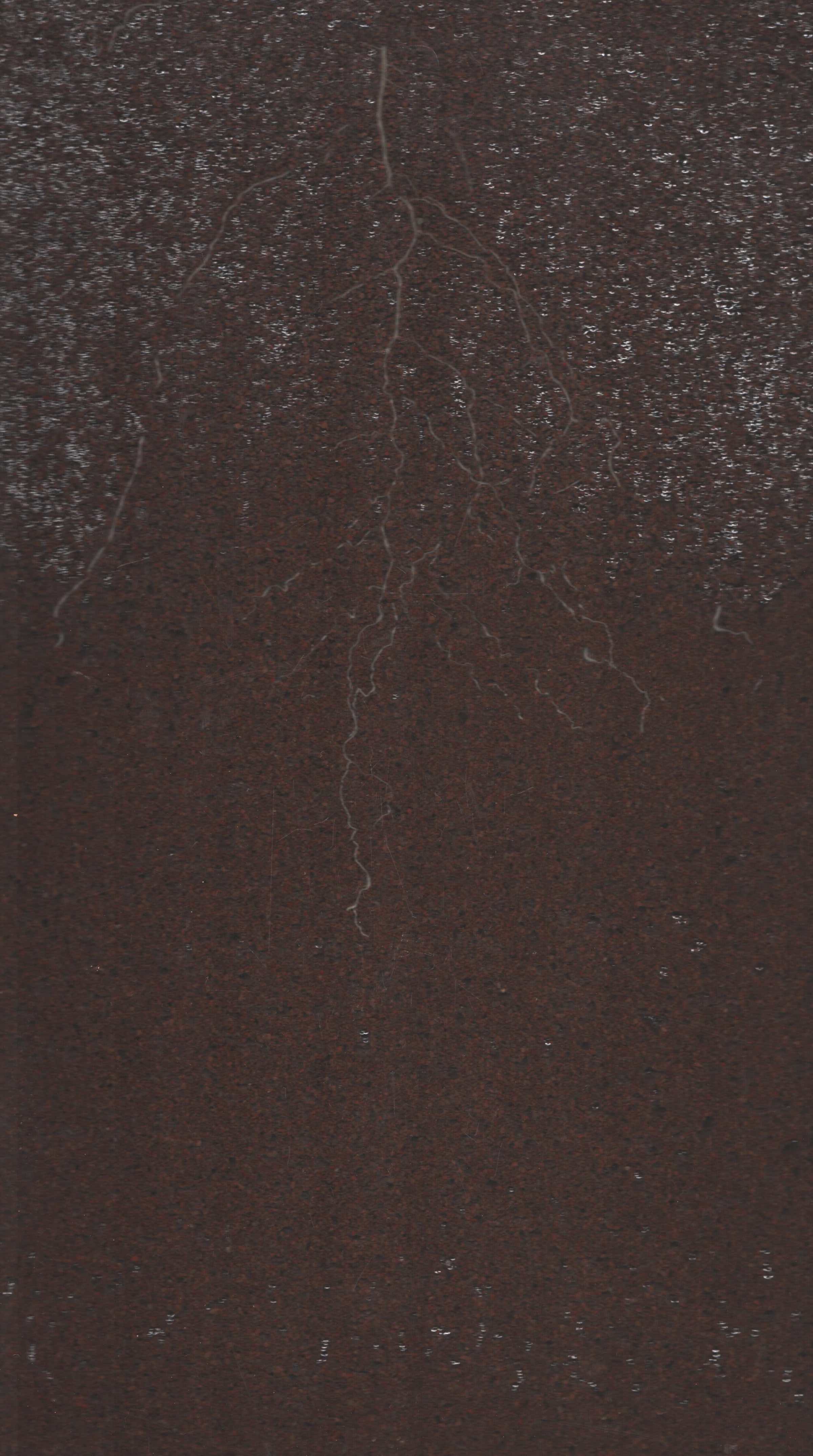

Supplement: Supplementary file 4 — Additional file 4: (a) Water level 60% (b) Water level 80% (c) Water level 100% (d) Water level 120% Fig. S4: RGB root images of the same cultivars (S38) with different water level treatments. [file 13007_2022_974_MOESM4_ESM.zip › FigureS4d.jpg]
